# Irreversible NADH Cleavage by a Mitochondria-Targeted Metal-Free Photoredox Sensitizer with Activity in Cancer Cells

**DOI:** 10.1021/acsmedchemlett.6c00205

**Published:** 2026-07-02

**Authors:** Jean C. Neto, Eva Falomir, Juan F. Miravet, Francisco Galindo

**Affiliations:** Departamento de Química Inorgánica y Orgánica, 456141 Universitat Jaume I de Castellón, Avda. Vicente Sos Baynat s/n, 12071 Castellón de la Plana, Spain

**Keywords:** metal-free photosensitizer, mitochondrial targeting, NADH cleavage, photobiological activity, cancer phototherapy, apoptosis

## Abstract

Intracellular photoredox catalysis has emerged as an alternative to classical photodynamic therapy, yet most systems promote reversible NADH oxidation to NAD^+^. Here we show that the mitochondria-targeted, metal-free organic photosensitizer **Cleav-1** induces photoinduced irreversible cleavage of NADH with nicotinamide release. **Cleav-1** is accessible in two steps, highly fluorescent, photostable, and efficiently accumulates in mitochondria of MCF-7 breast cancer cells. In an aqueous solution, it catalyzes light-driven NADH consumption, and ^1^H NMR detects free nicotinamide, supporting cofactor cleavage rather than redox interconversion. Consistent with a photoredox pathway, its first singlet excited state is efficiently quenched by NADH, whereas no singlet oxygen emission is observed. In cells, photoactivation of **Cleav-1** causes marked photocytotoxicity (IC_50_ ∼ 300 nM), severe depletion of intracellular NAD, mitochondrial dysfunction, and predominantly apoptotic cell death. These results suggest irreversible nicotinamide cofactor damage as a distinct photobiological mechanism and highlight mitochondria-targeted organic photoredox sensitizers for imaging-guided phototherapy.

## Graphical abstract

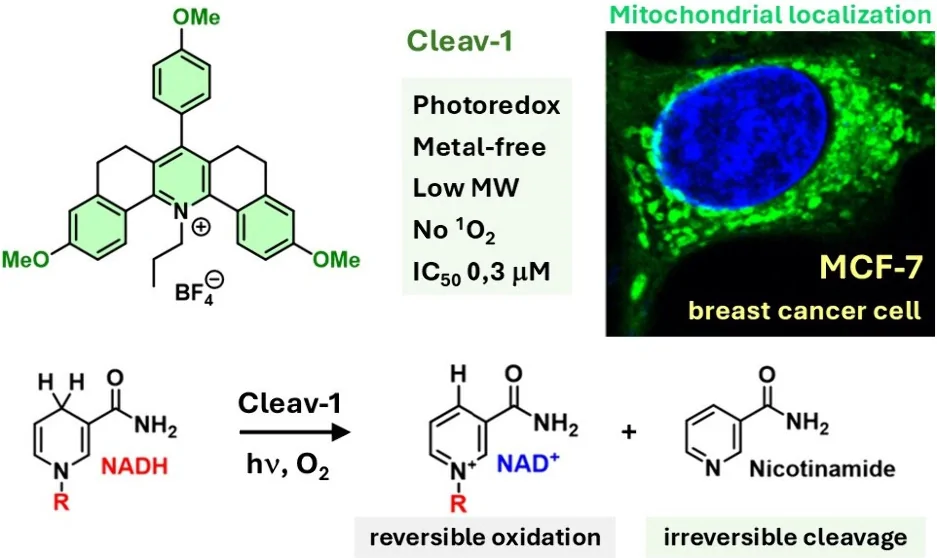

Light-driven therapy has a long history in both cancer treatment and antimicrobial applications, traditionally relying on photosensitizers that act through singlet oxygen or superoxide generation.^1–11^ In recent years, however, intracellular photoredox (PR) catalysis has emerged as an alternative framework for light-driven therapy, particularly under conditions where classical oxygen-dependent pathways are less effective. This concept, often referred to as photocatalytic therapy (PCT), has been explored with both metal-containing^12–25^ and, less frequently, metal-free photosensitizers.^26–31^ Across these systems, oxidation of reduced nicotinamide adenine dinucleotide (NADH) to nicotinamide adenine dinucleotide (NAD^+^) has become one of the benchmark reactions for assessing intracellular PR activity (other involve amino acids, DNA and lipid oxidations). In many cases, however, the observed photochemistry reflects a mixture of mechanisms, in which PR events coexist with conventional type I and type II pathways involving electron or energy transfer to O_2_.

Oxidation of NADH to NAD^+^ lies at the center of this emerging therapeutic concept because perturbation of the NADH/NAD^+^ couple can compromise cellular redox homeostasis, particularly in mitochondria, where oxidative phosphorylation depends critically on a continuous supply of reducing equivalents.^32^ This phenomenon, commonly described as photoinduced redox imbalance, is thought to contribute significantly to photocytotoxicity, especially under hypoxic conditions. Yet reversible oxidation of NADH to NAD^+^ does not necessarily constitute irreversible metabolic damage, since the redox balance can in principle be restored by endogenous NAD-dependent dehydrogenases.^33^ A substantially more severe outcome would arise if the reduced nicotinamide cofactor were not merely oxidized but irreversibly cleaved into a structurally different product. Unlike reversible perturbation of the NADH/NAD^+^ redox pair, irreversible cofactor cleavage depletes the functional nicotinamide pool and therefore requires de novo or salvage resynthesis for recovery (Figure 1). Related forms of irreversible NAD^+^ consumption are well precedented in biology, for example in PARP hyperactivation,^34^ SARM1 activation,^35^ and CD38 NADase activity.^36^

**Figure 1:**
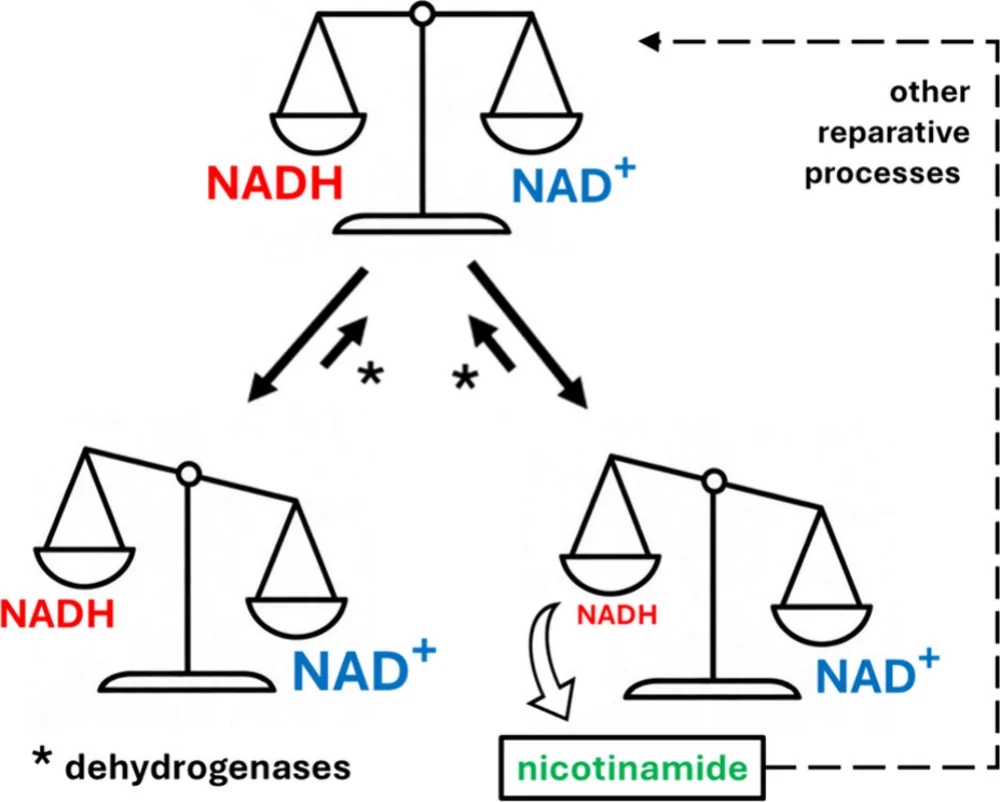
Photoinduced redox imbalance can be compensated by endogenous dehydrogenases unless it proceeds through an irreversible process, such as NADH cleavage with nicotinamide release.

Here we show that **Cleav-1** (Figure 2a), a small organic photooxidant with strong mitochondrial localization, induces irreversible photooxidative cleavage of NADH with release of nicotinamide (NIC) through C–N bond scission. In addition to in-cuvette photochemical experiments, cellular studies in MCF-7 breast cancer cells revealed marked photocytotoxicity, identifying this cationic chromophore as an efficient metal-free photosensitizer operating through a PR mechanism. Because the key feature of this process is that NADH consumption cannot be reversed by simple redox re-equilibration, we designate it as *photoinduced irreversible cleavage* (PIC), to distinguish it from previously described PR-induced redox imbalance. Notably, **Cleav-1** also stands out by operating through a single dominant pathway, as neither parallel energy transfer nor direct electron transfer to O_2_ makes a significant contribution under the conditions examined; instead, the photocatalytic cycle proceeds only in the presence of an oxidizable substrate.

**Figure 2:**
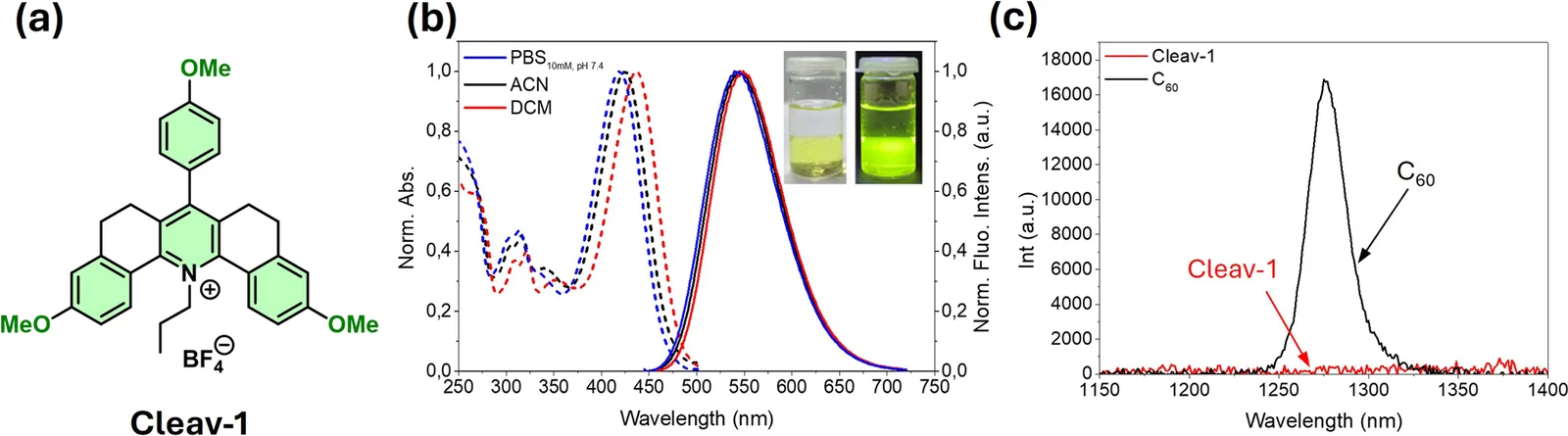
(a) Structure of photosensitizer **Cleav-1**; (b) Absorption and emission spectra of **Cleav-1** in PBS (10 mM, pH 7.4), acetonitrile and dichloromethane (inset: biphasic system with water and dichloromethane illustrating the lipophilicity of the cation, under visible and UV light); (c) Phosphorescence spectrum of singlet oxygen generated by fullerene C60 in toluene along with the flat signal recorded with irradiated **Cleav-1** in acetonitrile under identical excitation conditions.

**Cleav-1** belongs to a broader family of organic cationic chromophores related to triarylpyrylium derivatives used in photocatalysis^37,38^ and related to other established photoredox scaffolds.^39^ More broadly, the present work highlights the untapped potential of PIC not only for the development of new photosensitizers based on photoredox principles, but also for the reappraisal of existing organic dyes that are currently used primarily in synthetic photoredox catalysis.^26^

From a practical standpoint, an attractive feature of triarylpyridinium salts is their straightforward synthetic accessibility: they can be obtained in only two synthetic steps from commercially available starting materials and purified by simple precipitation.^40^ The dye object of this study, along with 19 related cationic derivatives, has recently been examined for intracellular organelle targeting in order to establish structure - property relationships relevant to bioimaging applications.^41^ Therefore, **Cleav-1** used here was prepared as described in this recent publication, and synthetic/characterization details of it can be found there.^41^

Photophysically, **Cleav-1** is highly emissive in both polar and apolar media, with fluorescence quantum yields (QY) of 0.70 in PBS (10 mM, pH 7.4), 0.76 in acetonitrile, and 0.89 in dichloromethane. This behavior likely arises from the conformational rigidity imposed by the carbon bridges linking rings 2 and 6, together with the electron-donating methoxy substituents. The high fluorescence efficiency suggests limited intersystem crossing to the first triplet state (T_1_) and therefore points to the first singlet excited state (S_1_) as the main reactive excited manifold. The compound is also lipophilic, with an estimated cLogP value of 5.75. Figure 2b shows its emission spectra in PBS, acetonitrile, and dichloromethane, together with a photograph of a biphasic water/DCM system illustrating its affinity for hydrophobic environments. The possible involvement of singlet oxygen after excitation was examined by monitoring the characteristic phosphorescence of ^1^O_2_ at 1270 nm and comparing the response with that of fullerene C_60_ in toluene as a reference. As shown in Figure 2c, no detectable ^1^O_2_ emission was observed under our conditions, indicating that this species does not make a significant contribution to the oxidative reactivity of this system. The main photophysical parameters of **Cleav-1** are summarized in Table 1.

**Table 1:** Photophysical Properties of Photosensitizer **Cleav-1** ^a^

Solv.	λ_abs_ (nm)	log ε	λ_fluo_ (nm)	Stokes shift (cm^–1^)	Stokes shift (nm)	Φ_f_	τ (ns)	*k*_r_ (10^7^ s^–1^)	*k*_nr_ (10^7^ s^–1^)
A^a^	424	4.13	546	5270	122	0.76	6.14	12	4
D^b^	436	4.49	548	4688	112	0.89	5.61	16	2
P^c^	419	4.41	543	5450	124	0.70	5.84	12	5

a Data reported in ref 41; λ_abs_ and λ_fluo_: absorption and emission maxima, respectively; log ε: logarithm of the molar absorption coefficient; Stokes shift expressed in wavenumbers and nanometers; Φ_f_: fluorescence quantum yield; τ: fluorescence lifetime; *k*_r_ and *k*_nr_: radiative and non-radiative rate constants, respectively.

b Acetonitrile.

c Dichloromethane.

d PBS (10 mM, pH 7.4).

The photosensitized oxidation of NADH mediated by **Cleav-1** was investigated following standard protocols commonly used in this field, particularly those established in the pioneering work of Sadler and co-workers.^12^ Thus, an aqueous solution of NADH (200 μM) in the presence of **Cleav-1** (5 μM) was irradiated for 40 min. As shown in Figures 3a and 3b, the progressive disappearance of the characteristic NADH absorption band at 339 nm reveals efficient substrate consumption, corresponding to an apparent rate constant of ca. 5.7 × 10^–2^ min^–1^ (Figure S1). Two commonly used descriptors in this context are the turnover number (TON) and turnover frequency (TOF). For the present system, the calculated TON and TOF were 36 and 53 h^–1^, respectively. A notable feature of **Cleav-1**, however, is its excellent photostability: the photosensitizer remained essentially unchanged after the irradiation period, as shown in the inset of Figure 3a and in the upper trace of Figure 3b. Although the system has not yet been optimized, this behavior suggests that additional oxidation cycles could in principle be sustained upon further addition of NADH, a point that will be explored in future studies.

**Figure 3:**
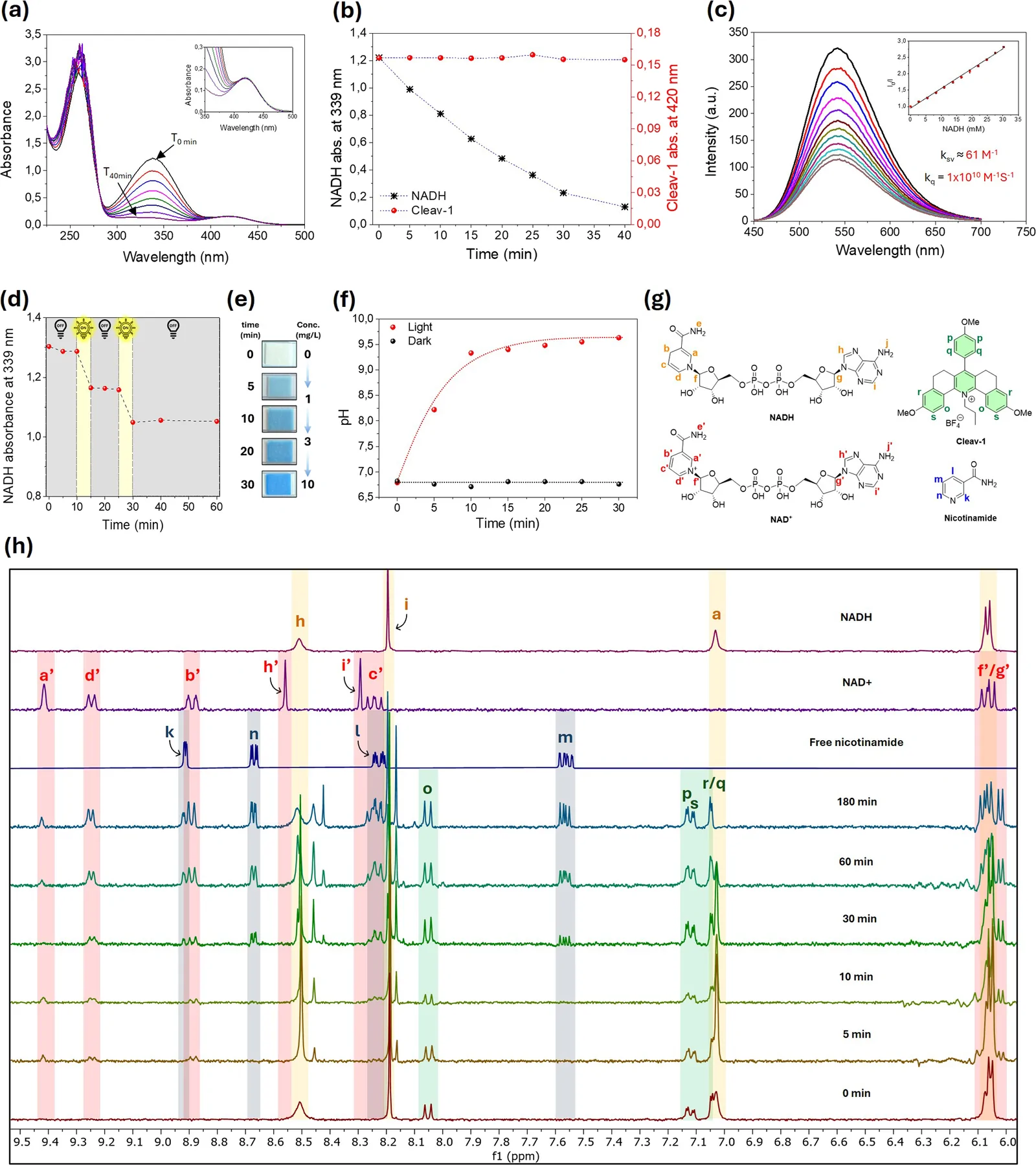
Oxidation and cleavage of NADH photosensitized by **Cleav-1**. (a) Time-dependent UV–Vis spectral evolution during photoirradiation of NADH (200 μM) in the presence of **Cleav-1** (5 μM) in PBS (10 mM, pH 7.4); insert: absorption region of the photocatalyst monitored throughout irradiation. (b) Evolution of NADH and **Cleav-1** absorbance as a function of time. (c) Stern–Volmer analysis of the fluorescence of **Cleav-1** (5 μM) recorded upon incremental addition of NADH (0–30 mM) in PBS (10 mM, pH 7.4); inset: Stern–Volmer plot (I_0_/I vs [NADH]) (d) Photoirradiation “on/off” experiment monitored at 339 nm. (e) H_2_O_2_ detected using peroxide indicator strips. (f) pH variation of the solution under light and dark conditions. (g) Chemical structures of NADH, NAD^+^, nicotinamide and **Cleav-1** with proton assignments. (h) Time-resolved (0 to 180 min) ^1^H NMR spectra recorded during photoirradiation of NADH (3 mM) in the presence of **Cleav-1** (0.25 mM) in H_2_O/D_2_O/CD_3_OD-d_4_ (0.5/49.5/50, v/v) containing NaCl (4 mM) and comparison with free NADH, NAD^+^ and nicotinamide.

Another distinctive feature of this photosensitizer is that the observed reactivity appears to arise predominantly from S_1_. This conclusion is consistent with its high emission QY and correspondingly low expected intersystem crossing and is further supported by Stern–Volmer quenching experiments. As shown in Figure 3c, the emission of **Cleav-1** is efficiently quenched by NADH, with a Stern–Volmer constant of K_SV_ = 61 M^–1^. Using a fluorescence lifetime of 5.8 ns, this corresponds to a bimolecular quenching constant of ca. 10^10^ M^–1^ s^–1^, i.e., a value close to the diffusion-controlled limit in water, consistent with highly efficient collisional quenching of S_1_.

The depletion of NADH is strictly light-dependent, as illustrated by the on/off irradiation experiment shown in Figure 3d. In parallel, the overall process is accompanied by the formation of hydrogen peroxide (H_2_O_2_, Figure 3e) and by an increase in pH, reaching values of approximately 9, as shown in Figure 3f.

Up to this point, **Cleav-1** behaves as a typical photoredox photosensitizer capable of catalyzing NADH oxidation. The key novelty of the present work, however, emerges from the ^1^H NMR analysis, which reveals a secondary photochemical transformation not previously described for related systems. Figure 3h shows the ^1^H NMR spectra recorded during irradiation of NADH in the presence of **Cleav-1**. At early reaction times, the first detectable process is the oxidation of NADH to NAD^+^, which is already evident within the first 5 min. At longer irradiation times, however, new resonances appear at ca. 7.6, 8.7, and 8.9 ppm. These signals match those of free nicotinamide (hereafter NIC, Figure 3g), as confirmed by direct comparison with an authentic commercial sample (see in the upper part of the Figure 3h, the ^1^H NMR spectra of NIC, NAD^+^ and NADH as references). Also, it is noteworthy the lack of **Cleav-1** photodegradation even after 3 h of irradiation.

This finding is significant and may have important biological implications because, to date, photosensitizers reported to operate through PCT have been primarily described as promoting oxidation of NADH to NAD^+^, rather than cleavage of the cofactor itself. The process described here instead entails a much more drastic transformation, one that cannot be readily reversed by cellular enzymatic machinery. As discussed earlier, several biological processes involving irreversible NAD^+^ consumption share this same conceptual feature: once the nicotinamide cofactor architecture has been fragmented, recovery no longer depends on simple redox re-equilibration, but instead requires cofactor resynthesis. To the best of our knowledge, only Xia and co-workers have described a partially related system.^42^ In that case, however, the photosensitizer was a conjugated polymer and the process was reductive rather than oxidative, involving electron injection into NAD^+^, followed by cofactor cleavage.

From a mechanistic standpoint, the photocatalytic cycle can be depicted as shown in Figure 4. Photoexcitation of the photosensitizer from the ground state generates the S_1_ excited state, from which single-electron transfer (SET) from NADH is proposed to occur, yielding the corresponding radical cation NADH^·+^. This unstable intermediate is expected to undergo deprotonation to afford the well-documented NAD· radical. This species occupies a central position in the mechanism. According to the conventional view, NAD· undergoes rearomatization to give NAD^+^, with participation of superoxide radical anion and protons. This pathway is also likely to be operative in our case, as supported by the detection of NAD^+^ by ^1^H NMR, along with the observed increase in pH and the formation of H_2_O_2_. However, fragmentation of NAD· to yield NIC and an ADP-ribose radical represents a competitive pathway that is compatible with our experimental observations. Direct photooxidation of NAD^+^ by **Cleav-1** to give NIC can be excluded on the basis of an independent control experiment (see Supporting Information, Figure S2).

**Figure 4:**
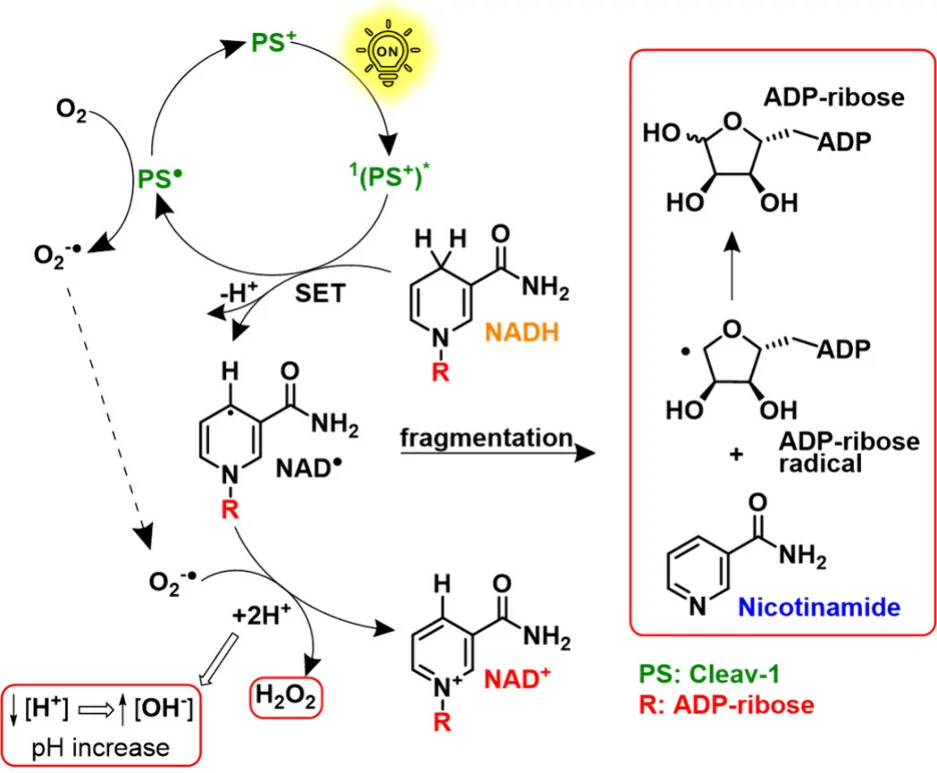
Mechanistic proposal for **Cleav-1**-mediated NADH photooxidation and cleavage.

The feasibility of NAD· cleavage is supported by previous studies in two different contexts, namely direct UV photolysis^43^ of NADH and photocatalysis with semiconductor materials.^44^ In the latter case, the branching between NAD^+^ formation and nicotinamide-releasing fragmentation appears to depend primarily on the oxidation mode of NADH: radical-mediated stepwise electron–proton–electron transfer favors fragmentation, whereas mediated one-step hydride transfer strongly shifts the reaction toward NAD^+^. In studies based on direct UV irradiation, fragmentation has been proposed to be favored under oxygen-poor conditions. A detailed mechanistic dissection of the present system lies beyond the scope of this work and will be addressed in future studies. Here, we focus instead on the photoredox activity of **Cleav-1** at the biological level.

**Cleav-1** was incubated with MCF-7 breast cancer cells for 30 min at a concentration of 100 nM, resulting in rapid cellular internalization and a punctate filamentous fluorescence pattern characteristic of mitochondrial networks, as observed by confocal laser scanning microscopy (CLSM) (Figure 5a). Co-staining with the commercial mitochondrial marker MitoTracker Deep Red (MTDR) confirmed predominant mitochondrial localization, consistent with the lipophilic cationic character of the probe and the well-established electrophoretic accumulation of similar probes.^45,46^ Quantitative colocalization analysis gave a high Pearson’s correlation coefficient (PCC = 0.87, Figure 5b), indicative of strong spatial correlation between **Cleav-1** and MTDR. Fluorescence intensity profiles (line-scan analysis) across selected regions of interest further showed close overlap between probe and tracker signals along mitochondrial structures (Figure 5c), further supporting mitochondrial targeting. Such localization is particularly advantageous for photodynamic and photoredox-based therapeutic strategies, as mitochondria are central to cellular bioenergetics, redox homeostasis, and apoptotic signaling.

**Figure 5:**
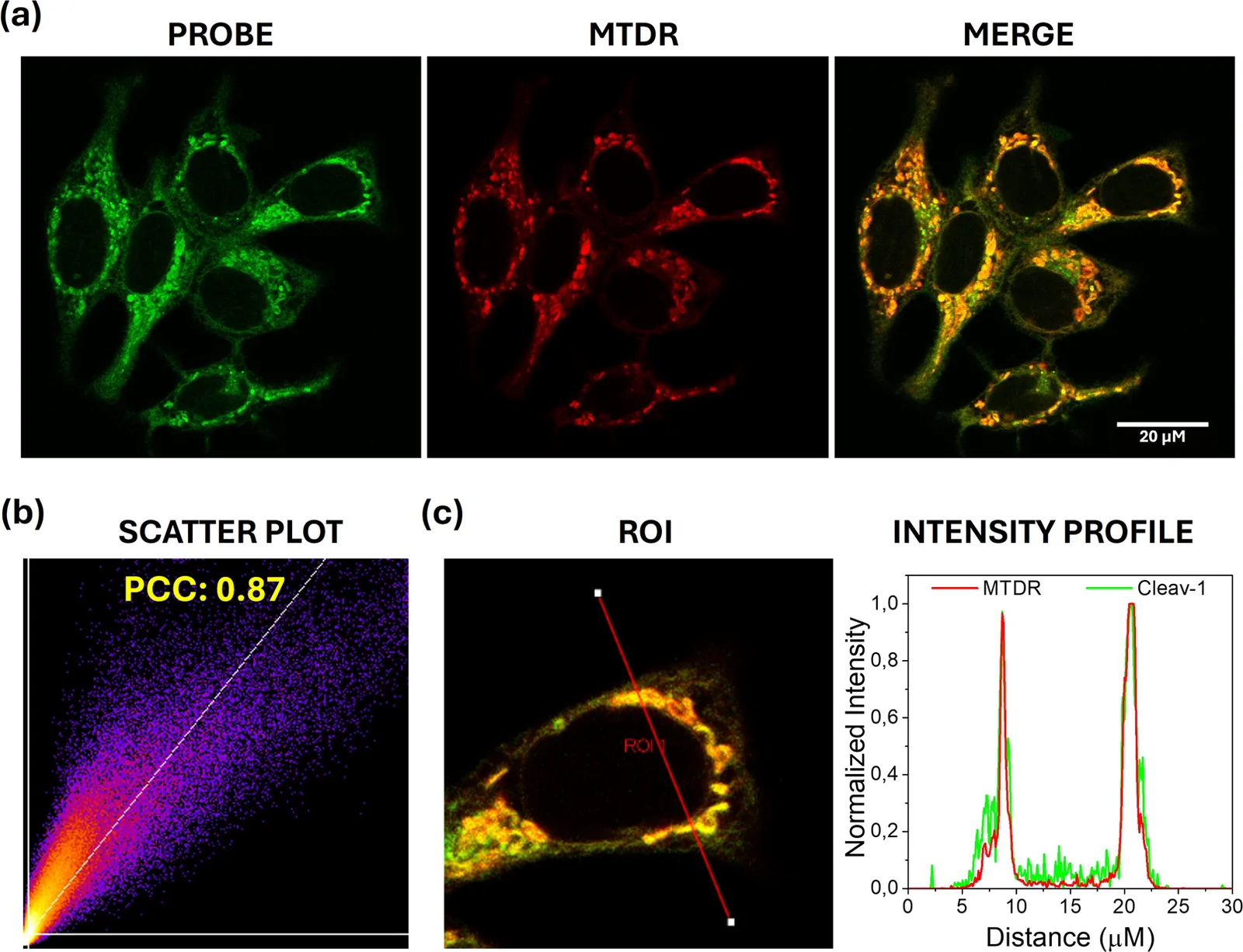
Confocal laser scanning microscopy (CLSM) analysis of intracellular localization. (a) MCF7 cells incubated with 100 nM of **Cleav-1** and Mitotracker Deep Red (MTDR) for 30 min at 37 °C. Scale bar: 20 μm. Green channel: excitation with 458 nm laser (**Cleav-1**); red channel: excitation with 633 nm laser (Mitotracker Deep Red FM). Also shown the overlay of green and red channels (merge). (b) Pixel-by-pixel fluorescence intensity correlation analysis between the probe and MTDR channels, with the corresponding scatter plot and Pearson’s correlation coefficient (PCC). (c) Region of interest (ROI) selected from the merged image (left) and the corresponding fluorescence intensity line profile (right), showing normalized fluorescence intensity of the probe (green) and MTDR (red) as a function of distance along the indicated line.

The phototherapeutic activity of **Cleav-1** was evaluated in human breast adenocarcinoma MCF-7 cells. Dose–response experiments revealed a strongly light-dependent cytotoxic effect, with IC_50_ values of 38.6 μM in the dark and 0.3 μM under irradiation (Figure 6a). These values correspond to a phototoxicity index (PI = IC_50_^dark^/IC_50_^light^) of approximately 130, indicating high light responsiveness together with low intrinsic toxicity in the absence of irradiation. In comparative terms, this level of photoresponse is consistent with that reported for representative phototherapeutic systems operating through PCT-type mechanisms.

**Figure 6:**
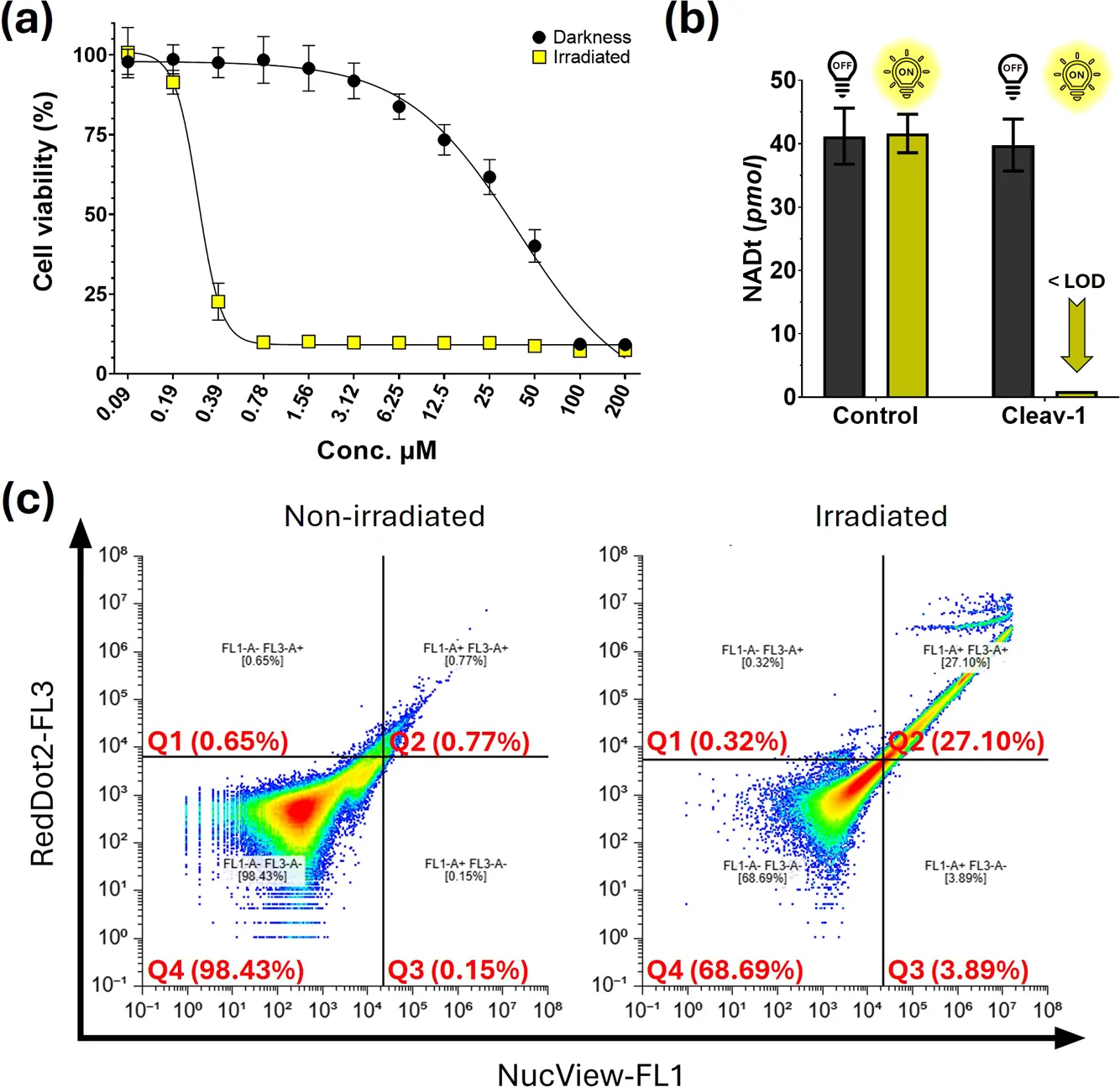
Photodynamic activity of **Cleav-1** in MCF-7 cells. (a) Cell viability of MCF-7 cells treated with increasing concentrations of **Cleav-1** under dark and irradiated conditions. Light exposure was performed using white light irradiation (see Supporting Information for details). (b) Intracellular total NAD levels (NADt = NADH + NAD^+^) quantified in cells treated with **Cleav-1** (2 μM) under dark and irradiated conditions. Values below the limit of detection (LOD) are indicated. (c) Flow cytometry analysis of apoptosis and necrosis using NucView (FL1, caspase activation marker) and RedDot2 (FL3, membrane integrity marker). Quadrant distribution: Q1 (NucView^–^/RedDot2^+^) necrotic cells; Q2 (NucView^+^/RedDot2^+^) late apoptosis; Q3 (NucView^+^/RedDot2^–^) early apoptosis; Q4 (NucView^–^/RedDot2^–^) viable cells.

To assess whether this phototoxic response was associated with metabolic perturbation, intracellular total NAD levels (NADt = NADH + NAD^+^) were quantified (Figure 6b). Whereas control cells and cells exposed to light alone retained detectable NADt levels, irradiation of cells previously incubated with **Cleav-1** led to a marked decrease in intracellular NADt, reaching values below the detection limit of the assay. This observation is mechanistically significant, because simple oxidation of NADH to NAD^+^ would be expected to alter the NADH/NAD^+^ ratio without necessarily causing major depletion of the total cofactor pool. The observed loss of NADt is therefore consistent with a substantial disruption of intracellular NAD homeostasis that extends beyond mere redox interconversion. It is worth noting that, besides NADH, mitochondria contain other potentially oxidizable substrates. Therefore, further studies are needed to determine the extent of oxidative damage and identify the specific targets affected.

The mechanism of cell death was next investigated by flow cytometry using NucView, a marker of caspase activation, and RedDot2, a marker of membrane integrity (Figure 6c). Untreated cells were located predominantly in the viable quadrant (NucView^–^/RedDot2^–^). By contrast, cells treated with **Cleav-1** and subsequently irradiated showed a marked increase in the NucView-positive population, consistent with caspase activation and apoptotic cell death, whereas only a minor fraction corresponded to primary necrosis (approximately 30% total apoptosis, with ∼ 0.3% classified as primary necrotic events). These results indicate that photoactivation of **Cleav-1** predominantly triggers programmed cell death rather than nonspecific membrane lysis.^47^

Confocal laser scanning microscopy (CLSM) provided complementary morphological evidence in support of this interpretation (Figure 7). Following irradiation in the presence of the photosensitizer, pronounced alterations in nuclear morphology were observed, including chromatin condensation and nuclear deformation, both characteristic features of apoptosis. In addition, the initially punctate mitochondrial staining pattern became more diffuse after light exposure, suggesting mitochondrial dysfunction. The presence of membrane blebbing further supports an apoptotic phenotype. Collectively, the viability data, NAD depletion measurements, and cytometric and morphological analyses indicate that light-activated **Cleav-1** induces severe disruption of intracellular redox homeostasis, metabolic impairment (likely associated to biomolecule oxidation), and predominantly apoptotic cell death in MCF-7 cells.

**Figure 7:**
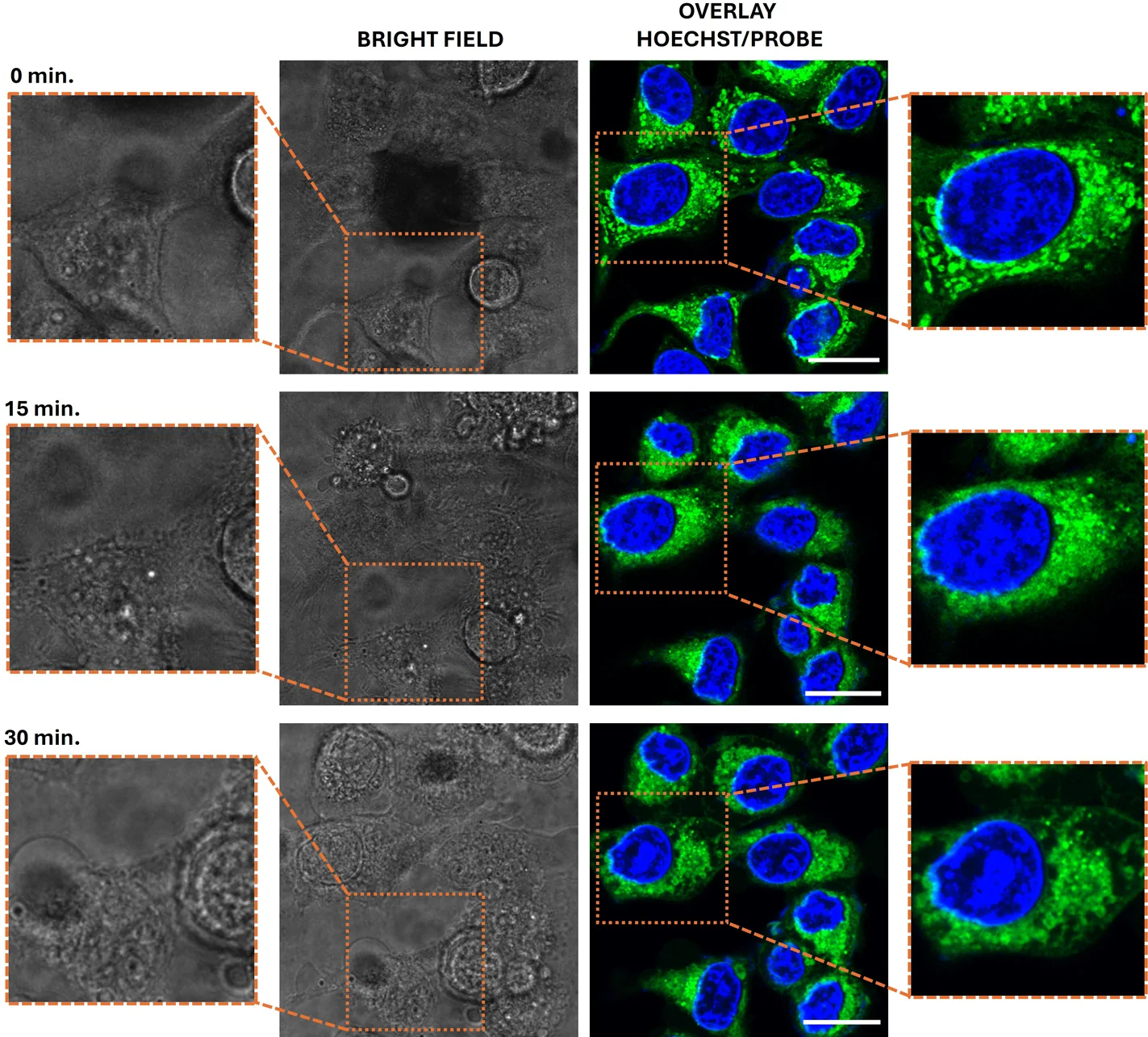
Confocal laser scanning microscopy (CLSM) images of MCF-7 cells incubated with **Cleav-1** (1 μM) and Hoechst 33342 for 30 min at 37 °C. Bright-field (left) and merged fluorescence images (right) are shown at different irradiation times (0, 15, and 30 min). Blue channel corresponds to nuclei stained with Hoechst 33342 (λ_exc_ 405 nm), and the green channel corresponds to **Cleav-1** (λ_exc_ 458 nm). Zoomed-in regions are highlight morphological changes and intracellular distribution. Cells were irradiated with white light (see Supporting Information for details). Scale bars: 20 μm.

The strong mitochondrial localization of **Cleav-1** is not merely a targeting feature but a therapeutically meaningful design element. Mitochondria are central regulators of cellular bioenergetics, redox balance, and apoptotic signaling, and their selective photodamage is therefore expected to amplify the biological consequences of photoactivation. In this context, mitochondrial targeting should be viewed as a directed therapeutic strategy rather than a simple localization phenomenon, because it concentrates photochemical reactivity at a particularly vulnerable intracellular site while minimizing inefficient off-target distribution.^1^ Such spatial control is especially valuable for photoredox-based systems, in which local cofactor perturbation and redox disruption may translate rapidly into mitochondrial dysfunction and cell death.

An additional advantage of **Cleav-1** is its high fluorescence quantum yield together with its marked photostability under the conditions employed. Because the photosensitizer remains strongly emissive throughout the process, it can be visualized during cellular uptake, subcellular localization, and photoinduced response without substantial signal loss from photobleaching. This combination of therapeutic activity and persistent fluorescence is attractive from the perspective of imaging-guided therapy,^1^ as it enables real-time tracking of the agent while preserving its photochemical function. More broadly, these features highlight the potential of this scaffold as a compact theranostic platform integrating photoredox activity with optical readout.

Although structurally related chalcogenopyrylium dyes have been studied previously in PDT, their photobiological activity was generally interpreted within a classical oxygen-dependent framework.^48^ This is not surprising, as thiopyrylium, selenopyrylium, and telluropyrylium derivatives were typically designed to exploit heavy-atom-induced intersystem crossing and enhanced singlet oxygen generation. In those systems, phototoxicity was therefore mainly associated with conventional type II mechanisms. By contrast, **Cleav-1** points to a different opportunity within this broader structural family: the development of low-ISC cationic chromophores that operate predominantly through photoredox chemistry. The irreversible NADH cleavage reported here suggests that pyrylium-derived photosensitizers may have underexplored potential as mechanistically distinct phototherapeutic agents beyond conventional PDT.

Looking ahead, it seems likely that several classical photosensitizers will be worth revisiting, with their biological mechanisms of action reinterpreted through the lens of photoinduced redox chemistry. In this context, Sarna and co-workers reported the photooxidative reactivity of Rose Bengal (RB) toward a range of electron donors, including NADH.^49^ Recent studies corroborated these observations and broadened their scope to additional, widely used photosensitizers.^26^ Complementarily, work from P. Klán’s group has demonstrated that RB can also promote the photooxidation of amino acids such as tryptophan and tyrosine,^50^ highlighting proteins as additional, chemically plausible targets for photoredox pathways.^2,20–22^

In summary, **Cleav-1** is a readily accessible, metal-free, mitochondria-targeted photoredox sensitizer that combines favorable photophysical properties, strong mitochondrial accumulation, and marked light-dependent activity in MCF-7 cells. Under irradiation, the compound efficiently promotes NADH consumption, while ^1^H NMR analysis reveals the formation of free nicotinamide, supporting an irreversible NADH cleavage pathway rather than simple NADH/NAD^+^ redox interconversion. This cleavage-centered reactivity represents the main conceptual advance of the present work, as irreversible cofactor fragmentation is expected to deplete the functional nicotinamide pool in a manner that cannot be readily compensated by endogenous redox recycling.

The biological profile of **Cleav-1** further highlights the relevance of this design. In cells, photoactivation leads to strong photocytotoxicity, severe depletion of total intracellular NAD, mitochondrial perturbation, and predominantly apoptotic cell death, consistent with a biologically disruptive photoredox process. In this context, mitochondrial localization should be viewed not merely as a targeting feature, but as a therapeutically meaningful strategy that concentrates photochemical reactivity at a particularly vulnerable intracellular site while minimizing off-target distribution. In addition, the high fluorescence quantum yield and pronounced photostability of **Cleav-1** allow persistent optical visualization during uptake, localization, and photoactivation, thereby adding an imaging component to its therapeutic function. More broadly, these results suggest that metal-free photosensitizers capable of promoting nicotinamide cofactor cleavage may open new opportunities for the development of mechanistically differentiated phototherapeutic and imaging-guided agents.

## Supplementary Material



## Data Availability

The data supporting the findings of this study are available from the corresponding author upon reasonable request.

## Notes

Experimental details, including kinetic analysis, NMR experiments, biological assays, and imaging methodologies (PDF)

## Notes

No unexpected or unusually high safety hazards were encountered

## Abbreviations

ADP: adenosine diphosphate
CLSM: confocal laser scanning microscopy
DCM: dichloromethane
H_2_O_2_: hydrogen peroxide
IC_50_: half-maximal inhibitory concentration
ISC: intersystem crossing
LOD: limit of detection
MTDR: MitoTracker Deep Red
NAD: nicotinamide adenine dinucleotide
NAD^+^: oxidized nicotinamide adenine dinucleotide
NADH: reduced nicotinamide adenine dinucleotide
NADt: total intracellular nicotinamide adenine dinucleotide
NIC: nicotinamide
PCC: Pearson’s correlation coefficient
PBS: phosphate-buffered saline
PCT: photocatalytic therapy
PIC: photoinduced irreversible cleavage
PI: phototoxicity index
PR: photoredox
QY: fluorescence quantum yield
RB: Rose Bengal
ROI: region of interest
ROS: reactive oxygen species
SET: single-electron transfer
S_1_: first singlet excited state
T_1_: first triplet excited state
TON: turnover number
TOF: turnover frequency
UV–Vis: ultraviolet–visible spectroscopy
^1^O_2_: singlet oxygen
