# Supplementary material for: Irreversible NADH Cleavage by a Mitochondria-Targeted Metal-Free Photoredox Sensitizer with Activity in Cancer Cells
Source: ACS Med Chem Lett. 2026 Jul 2;17(8):1806–15. doi: 10.1021/acsmedchemlett.6c00205 (PMC13488117; doi:10.1021/acsmedchemlett.6c00205)
Supplement: Supplementary file 1 [file ml6c00205_si_001.pdf]

Irreversible NADH Cleavage by a Mitochondria-Targeted Metal-Free Photoredox Sensitizer with Activity in Cancer cells

Jean C. Neto,[a] Eva Falomir,[a] Juan F. Miravet,[a] Francisco Galindo*[a]

[a] *Universitat Jaume I de Castellón, Departamento de Química Inorgánica y Orgánica, Avda. Vicente Sos Baynat s/n, 12071 Castellón de la Plana, Spain.*

* E-mail: francisco.galindo@uji.es

NADH oxidation monitored by UV–Vis spectroscopy 2

Stern–Volmer analysis 2

Light-dependent study 2

pH monitoring 3

Detection of H_2O_2 generation 3

^{1}H NMR studies. 3

Confocal Laser Scanning Microscopy (CLSM). 3

Dark- and photo-cytotoxicity 4

Determination of intracellular total NAD (NADt) 5

Cell death mechanism analysis 5

CLSM imaging under photoirradiation. 6

Figure S1. Kinetic analysis of NADH oxidation under irradiation in the presence of Cleav-1. 7

Figure S2. ^{1}H NMR spectra of NAD^+, photoirradiated NAD^+/Cleav-1, and nicotinamide 7

Supplementary references 8

NADH oxidation monitored by UV–Vis spectroscopy

The photoinduced oxidation of NADH was monitored by UV–Vis absorption spectroscopy following literature procedures reported with modifications.[1,2] Absorption measurements were carried out in PBS (10 mM, pH 7.4) using NADH (200 µM) and the photosensitizer **Cleav-1** (5 µM). Samples were irradiated using white light (TENKO ECO 50 W LED, 90 mW/cm², 420–700 nm) and the reaction was monitored from 0 to 40 min (5.4 J/cm^2 per min.). The conversion of NADH was followed by the decrease in absorbance at 339 nm over time, and NADH concentration was determined using the Beer–Lambert law with a molar extinction coefficient of $\varepsilon_{339} = 6220\ M^{-1}cm^{-1}$.[3] Catalytic parameters were calculated from these data: the turnover number (TON) was defined as the ratio between the amount of NADH consumed and the concentration of photosensitizer, while the turnover frequency (TOF) was obtained by dividing TON by the reaction time. First-order kinetic analysis was performed by plotting ln(A_{339}) versus irradiation time. The apparent rate constant (k) was obtained from the slope of the linear fit, and the half-life ($t_{1/2}$) was calculated as ln(2)/k.

Stern–Volmer analysis

The interaction between **Cleav-1** and NADH was investigated by Stern–Volmer analysis.[1,4] Emission spectra of **Cleav-1** were recorded in PBS (10 mM, pH 7.4) in the absence and presence of increasing concentrations of NADH (0–30 mM). The fluorescence intensity was measured upon excitation at 425 nm. The data were analyzed using the Stern–Volmer equation ($I_0/I = 1 + K_{SV}[Q]$), where I_0 and I correspond to the emission intensities in the absence and presence of NADH, respectively, and [Q] is the concentration of NADH. K_{SV} values were obtained from the slope of the linear fit of the Stern–Volmer plot (I_0/I vs [NADH]). The bimolecular quenching constant (k_q) was calculated using the relation $k_q = K_{SV}/\tau_0$, where τ_0 is the fluorescence lifetime of **Cleav-1** in the absence of quencher.

Light-dependent study

The light-dependent activation of **Cleav-1** was evaluated using alternating irradiation and dark cycles. Samples containing NADH (200 µM) and **Cleav-1** (5 µM) in PBS (10 mM, pH 7.4) were subjected to

successive periods of irradiation with white light and absence of light. The reaction was monitored by measuring the absorbance at 339 nm over time.

pH monitoring

The variation of pH during the photoinduced process was evaluated using NADH (200 µM) and **Cleav-1** (5 µM) in distilled water under irradiation with white light. pH values were recorded at defined time intervals using a Consort C860 pH meter.

Detection of H_2O_2 generation

Hydrogen peroxide formation was assessed for solutions containing **Cleav-1** (12.5 µM) and NADH (500 µM) in PBS (10 mM, pH 7.4). Detection was performed using MQuant® Peroxide-Test strips (Sigma), comparing samples kept in the dark and after irradiation with white light.

^{1}H NMR studies.

NADH (3 mM) was added to an NMR tube containing **Cleav-1** (0.25 mM) in a solvent mixture of $H_2O/D_2O/CD_3OD$-d$_4$ (0.5:49.5:50, v/v) supplemented with NaCl (4 mM).[1] The samples were prepared at ambient temperature and homogenized prior to analysis. ^{1}H NMR spectra were recorded at 310 K on a Bruker Avance III HD spectrometer operating at 400 MHz for ^{1}H at defined time intervals, including measurements at 0 min (dark), and after photoirradiation using a white light source for the specified time. Time-resolved spectra were acquired to monitor the evolution of NADH oxidation and the subsequent formation of NAD^+ and nicotinamide.

Confocal Laser Scanning Microscopy (CLSM).

The preparation of IBIDI plates for confocal microscopy was carried out following previously reported procedures.[5] MCF-7 cells were cultured in IBIDI 4-well plates and allowed to grow for 24 h at 37 °C in a humidified atmosphere containing 5% CO_2 to ensure proper adhesion. After the incubation period, the cells were washed three times with phosphate-buffered saline (PBS) to remove residual culture medium. The cells were then incubated with **Cleav-1** (100 nM, 30 min) and MitoTracker Deep Red FM (100 nM, 30 min) at 37

°C. After incubation, the staining solution was removed, and the cells were washed three times with PBS to eliminate unbound probes. Confocal images were acquired using a Leica TCS SP8 confocal microscope equipped with a 60x oil immersion objective. **Cleav-1** was excited using a 458 nm laser, and emission was collected in the 500–550 nm range. MitoTracker Deep Red was excited at 633 nm, with emission collected between 650–750 nm. Images for each channel were recorded separately and subsequently processed for analysis.

Dark- and photo-cytotoxicity

Cell viability was evaluated using the MTT assay, following previously reported procedures.[5] MCF-7 cells were seeded in 96-well plates at a density of approximately 5×10^4 cells per well and incubated in appropriate culture medium at 37 °C under a humidified atmosphere containing 5% CO_2 for 24 h to allow cell adhesion and growth. After this period, the culture medium was replaced and the cells were treated with **Cleav-1** at the desired concentrations, followed by incubation for 30 min under the same conditions. Subsequently, the plates were divided into two groups: one plate was irradiated using white light source under continuous orbital shaking, while the second plate was kept in the dark under identical conditions of temperature and time as a control. After irradiation, both plates were returned to the incubator and maintained for an additional 60 min. The culture medium was then carefully removed and replaced with fresh medium, followed by the addition of MTT solution (10 µL, 5 mg/mL in 1× PBS) to each well. The plates were incubated for 4 h at 37 °C to allow the formation of formazan crystals by metabolically active cells. After incubation, the medium was removed, and the formed formazan crystals were dissolved by adding 100 µL of dimethyl sulfoxide (DMSO) to each well. The plates were gently shaken for 10 min to ensure complete dissolution. Absorbance was measured using a microplate reader (Multiskan FC) at 570 nm, with a reference wavelength of 630 nm. Cell viability was expressed relative to untreated control cells, and IC_{50} values were determined by nonlinear regression analysis using GraphPad Prism software.

Determination of intracellular total NAD (NADt)

The quantification of total intracellular NAD (NADt, NAD^+ + NADH) was performed using a commercial colorimetric assay kit (ab65348, Abcam), following the manufacturer's instructions with minor modifications. MCF-7 cells were prepared under the same conditions described for the phototoxicity assays. Briefly, cells were seeded in 12-well plates, allowed to adhere for 24 h, and then treated with **Cleav-1** (2 μM) for 30 min. The plates were subsequently divided into two groups: one was irradiated with white light under orbital shaking, while the other was kept in the dark under identical conditions. After irradiation, both plates were returned to the incubator for an additional 60 min. Following treatment, cells were washed with cold PBS and processed for NAD extraction. Cell lysates were obtained using the extraction buffer provided in the kit, followed by freeze–thaw cycles and centrifugation to remove insoluble material. The supernatants were collected and, when necessary, filtered to eliminate interfering enzymes. For NADt determination, aliquots of the extracts were transferred to a 96-well plate, and the reaction was initiated by adding the NAD cycling reaction mixture according to the kit protocol. After a short incubation at room temperature, the developer solution was added, and the absorbance was monitored at 450 nm using a microplate reader (Multiskan FC) in kinetic mode. NADt concentrations were calculated from a standard calibration curve generated with NADH standards and expressed relative to control conditions.

Cell death mechanism analysis

Apoptosis and necrosis were evaluated using a commercial NucView® 488/RedDot™2 assay kit (Biotium), following the manufacturer's instructions with minor modifications. MCF-7 cells were prepared under the same conditions described for the phototoxicity assays. Briefly, cells were seeded, allowed to adhere for 24 h, and treated with **Cleav-1** (2 μM) for 30 min. The plates were then divided into two groups: one was irradiated with white light under orbital shaking, while the other was maintained in the dark under identical conditions. After irradiation, both plates were incubated for an additional 60 min. Following this period, the culture medium was removed, and the cells were washed with PBS, detached, pelleted, and washed three times with PBS. The staining procedure was then carried out according to the kit protocol. Briefly, cells were incubated with NucView® 488 caspase-3 substrate and RedDot™2 under light-protected conditions. Samples were

subsequently analyzed by fluorescence microscopy. NucView® 488 fluorescence (green channel) was used to detect caspase-3/7 activation (apoptotic cells), while RedDot™2 fluorescence (far-red channel) identified cells with compromised membrane integrity (necrotic or late apoptotic cells). Appropriate controls were included in all experiments, and the data were processed and analyzed using Floreada.io.

CLSM imaging under photoirradiation.

Live-cell imaging experiments were performed to monitor light-induced morphological changes over time. MCF-7 cells were prepared following the same procedure described for confocal microscopy studies. Briefly, cells were seeded in IBIDI 4-well plates, allowed to adhere for 24 h, and incubated with **Cleav-1** (1 µM, 30 min) and Hoechst 33342 for nuclear staining. After incubation, cells were washed three times with PBS to eliminate unbound probes. A region of interest was selected using a Leica TCS SP8 confocal microscope equipped with a 60× oil immersion objective. The selected area was continuously irradiated using white light (see previous description of the lamp), while sequential images were acquired at defined time intervals. Fluorescence images were recorded using a 405 nm laser for Hoechst 33342 (emission collected between 410–465 nm) and a 458 nm laser for **Cleav-1** (emission collected between 500–550 nm). Bright-field images were acquired in parallel. This time-resolved imaging approach enabled the visualization of progressive morphological changes, including cell shrinkage, membrane blebbing, and intracellular redistribution, induced by photoirradiation.

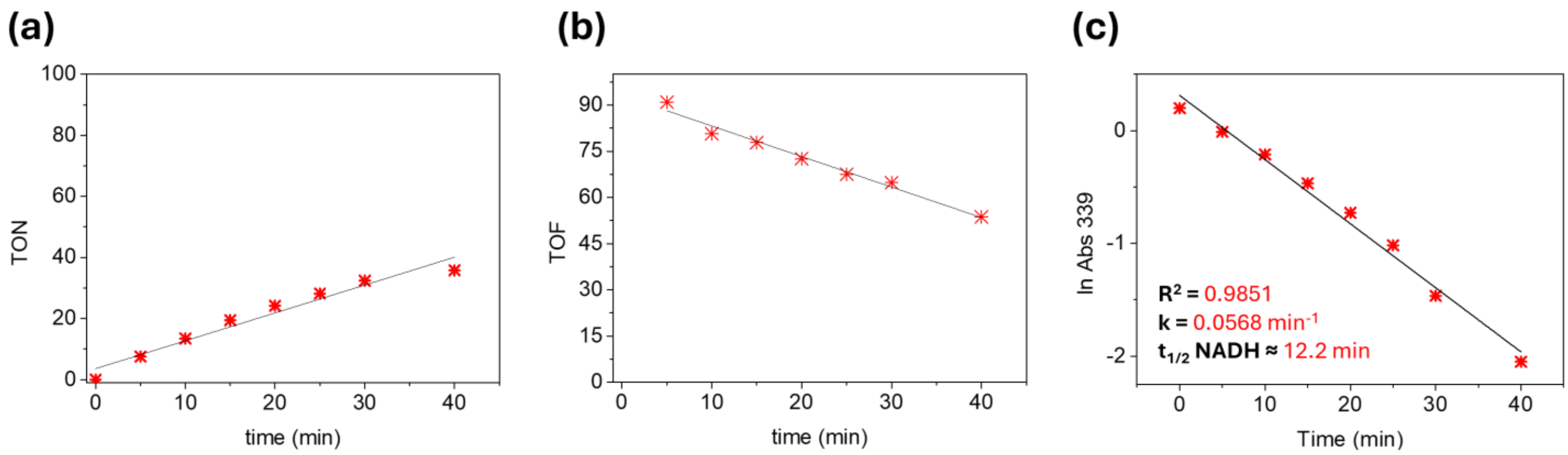

Figure S1. Kinetic analysis of NADH oxidation under irradiation in the presence of Cleav-1. Time-dependent evolution of TON (a) and TOF (b). (c) First-order kinetic plot of NADH consumption at 339 nm (ln Abs vs time), with linear fit (R^2), *k* (apparent first-order rate constant), and $t_{1/2}$ (half-life of NADH consumption).

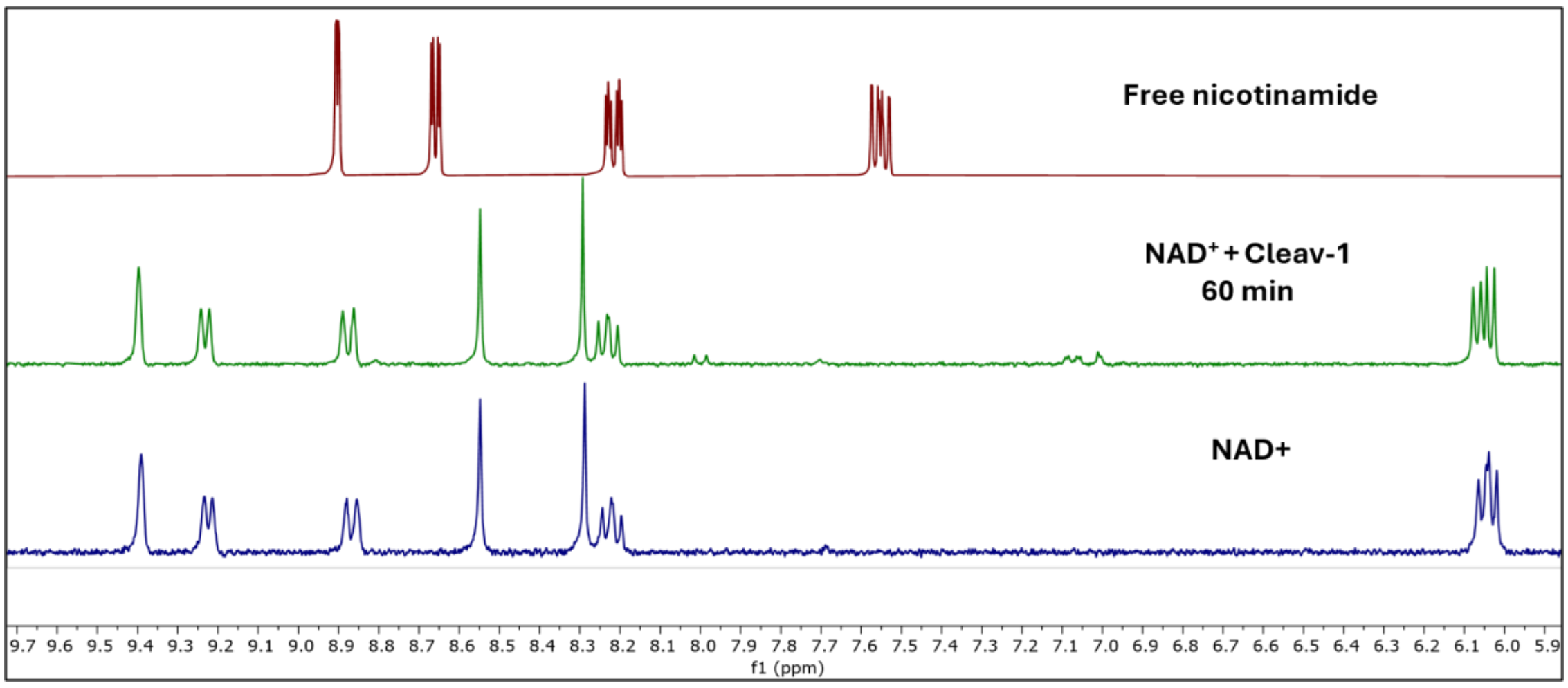

Figure S2. 1H NMR spectra of NAD+, photoirradiated NAD+/Cleav-1, and nicotinamide. Bottom, blue: ^{1}H NMR spectra of NAD^+; Middle, green: NAD^+ in the presence of **Cleav-1** irradiated with white light for 60 min; Top, red: free nicotinamide to illustrate that it is not produced after irradiation of NAD^+ and **Cleav-1**. All the spectra were acquired in $H_2O/D_2O/CD_3OD$-d_4 (0.5:49.5:50, v/v) containing NaCl (4 mM).

Supplementary references

(1) Huang, H.; Banerjee, S.; Qiu, K.; Zhang, P.; Blacque, O.; Malcomson, T.; Paterson, M. J.; Clarkson, G. J.; Staniforth, M.; Stavros, V. G.; Gasser, G.; Chao, H.; Sadler, P. J. Targeted Photoredox Catalysis in Cancer Cells. *Nat. Chem.* **2019**, *11* (11), 1041–1048. https://doi.org/10.1038/s41557-019-0328-4.

(2) Li, M.; Xu, Y.; Pu, Z.; Xiong, T.; Huang, H.; Long, S.; Son, S.; Yu, L.; Singh, N.; Tong, Y.; Sessler, J. L.; Peng, X.; Kim, J. S. Photoredox Catalysis May Be a General Mechanism in Photodynamic Therapy. *Proceedings of the National Academy of Sciences of the United States of America* **2022**, *119* (34), e2210504119. https://doi.org/10.1073/pnas.2210504119.

(3) Liu, Z.; Romero-Canelón, I.; Qamar, B.; Hearn, J. M.; Habtemariam, A.; Barry, N. P. E.; Pizarro, A. M.; Clarkson, G. J.; Sadler, P. J. The Potent Oxidant Anticancer Activity of Organoiridium Catalysts. *Angew Chem Int Ed Engl* **2014**, *53* (15), 3941–3946. https://doi.org/10.1002/anie.201311161.

(4) Beltrán, A.; Burguete, M. I.; Galindo, F.; Luis, S. V. Synthesis of New Fluorescent Pyrylium Dyes and Study of Their Interaction with N-Protected Amino Acids. *New J. Chem.* **2020**, *44* (22), 9509–9521. https://doi.org/10.1039/D0NJ02033H.

(5) Neto, J. C.; Lucantoni, F.; González, L. V.; Falomir, E.; Miravet, J. F.; Galindo, F. Introducing TAPY as a Versatile Alternative to TPP for Selective Mitochondrial Targeting in Cancer Cells. *Bioconjugate Chem.* **2025**, *36* (4), 697–706. https://doi.org/10.1021/acs.bioconjchem.4c00554.
